# Resolution-promoting autacoids demonstrate promising cardioprotective effects against heart diseases

**DOI:** 10.1007/s11033-022-07230-6

**Published:** 2022-02-10

**Authors:** Roddy Hiram

**Affiliations:** Department of Medicine, Faculty of Medicine, Montreal Heart Institute (MHI), Université de Montréal, Research Center, 5000 Belanger, St. Montreal, QC H1T 1C8 Canada

**Keywords:** Inflammation, Resolution, Cardiac diseases, Atrial Fibrillation, Resolvin

## Abstract

**Abstract:**

Chronic heart diseases have in common an unresolved inflammatory status. In atherosclerosis, myocarditis, myocardial infarction, or atrial fibrillation, mounting evidence suggests that unresolved inflammation contributes to the chronicity, aggravation, and morbidity of the disease. Following cardiac injury or infection, acute inflammation is a normal and required process to repair damaged tissues or eliminate pathogens and promote restoration of normal functions and structures. However, if acute inflammation is not followed by resolution, a chronic and deleterious inflammatory status may occur, characterized by the persistence of inflammatory biomarkers, promoting aggravation of myocardial pathogenesis, abnormal structural remodeling, development of cardiac fibrosis, and loss of function. Although traditional antiinflammatory strategies, including the use of COX-inhibitors, to inhibit the production of inflammation promotors failed to promote homeostasis, mounting evidence suggests that activation of specific endogenous autacoids may promote resolution and perpetuate cardioprotective effects. The recent discovery of the active mechanism of resolution suggests that proresolving signals and cellular processes may help to terminate inflammation and combat the development of its chronic profile in cardiac diseases. This review discussed (I) the preclinical and clinical evidence of inflammation-resolution in cardiac disorders including atrial fibrillation; (II) how and why many traditional antiinflammatory treatments failed to prevent or cure cardiac inflammation and fibrosis; and (III) whether new therapeutic strategies may interact with the resolution machinery to have cardioprotective effects.

**Graphical abstract:**

*RvD* D-series resolving, *RvE* E-series resolving, *LXA4* lipoxin A4, *MaR1* maresin-1
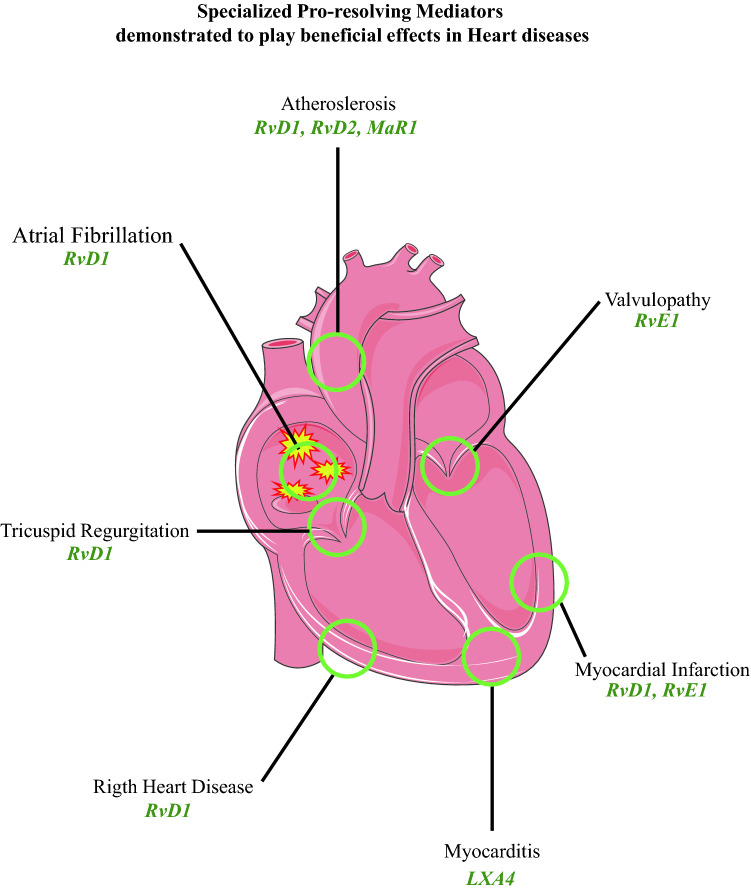

## Introduction

Unresolved inflammation is the common denominator of various cardiac disorders including myocarditis, atherosclerosis, congestive heart failure, and atrial arrhythmia [1]. Long-term use of traditional antiinflammatory medications is often responsible for various adverse effects and increased morbidity in patients with a history of cardiac disease [2]. Recent studies suggest that therapeutic strategies promoting the activation of recently described proresolving biological processes may contribute to attenuate the consequences of chronic inflammation in neurodegenerative diseases, pulmonary diseases, obesity, or aging [3]. Few data are available about the role of these specialized proresolving mediators (SPMs) in cardiac diseases.

**Fig. 1:**
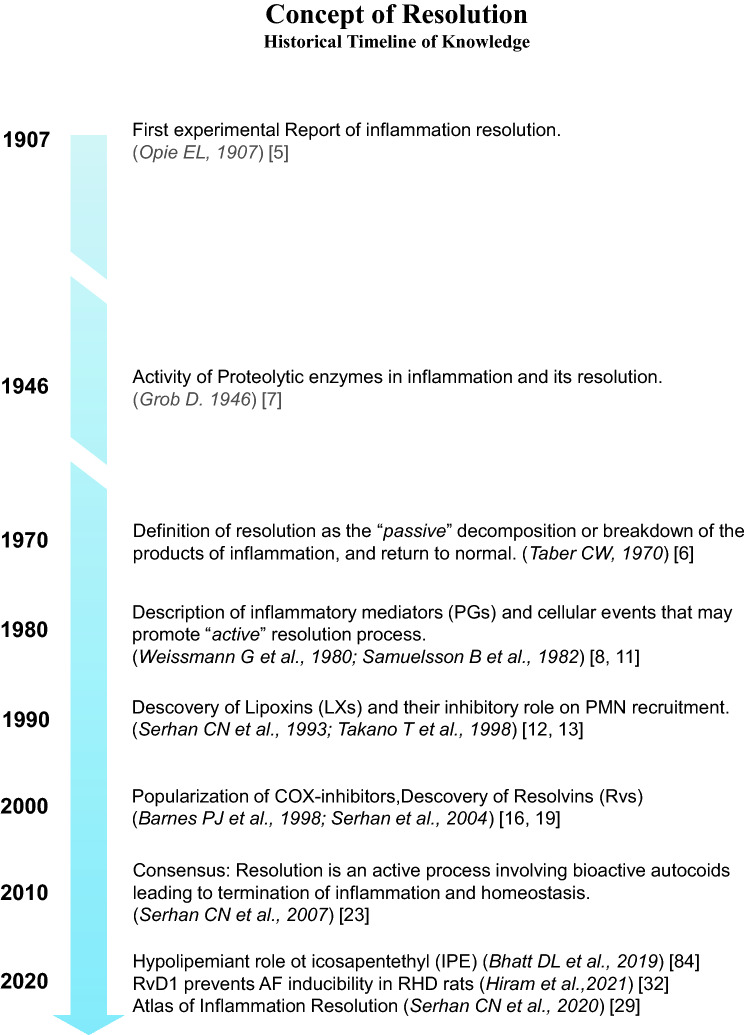
Concept of resolution: Historical timeline of knowledge. Evolution of the concept of resolution and significant discoveries from 1907 to 2021

**Fig. 2:**
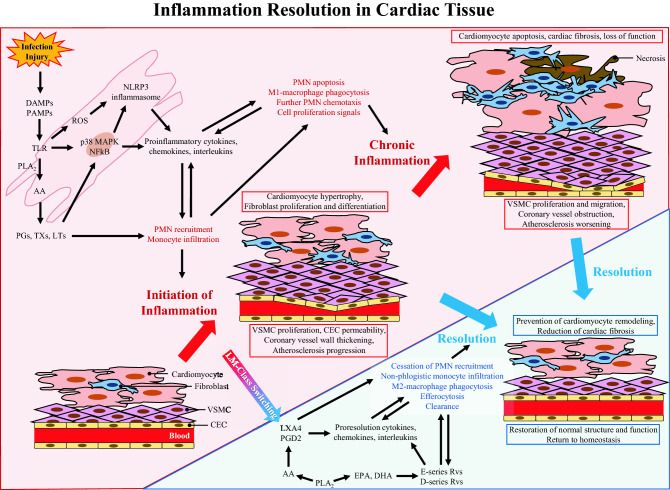
Inflammation resolution in cardiac tissue. Schematic of the vascular and tissue responses to inflammation after cardiac infection or injury. During acute inflammation, three consecutive phases aim to promote healing and homeostasis. Initiation: The initiation phase is characterized by increased proinflammatory signaling, PMN recruitment, and proinflammatory-(M1)-macrophage infiltration to phagocytize damaged cells and pathogens. LM-Class switching: Phagocytic PMN and M1-macrophages secrete 12/15 LOX enzyme. This promotes the lipid-mediator class switching, characterized by activation of secretion of proresolving lipids from AA, EPA, and DHA. These signals promote cessation of PMN infiltration and augmentation of anti-inflammatory-(M2)-macrophage phagocytosis of apoptotic PMN. Resolution: Complete resolution is identified by elimination of debris, efferocytosis, clearance, and restoration of normal cardiac function and structure. Chronic Inflammation: Failure in the activation of proresolution signals may promote chronic inflammation, characterized by the persistence of proinflammatory lipid mediators and proinflammatory cytokines, cardiomyocytes hypertrophy and necrosis, cardiac fibrosis, ischemia, and loss of cardiac function

**Fig. 3:**
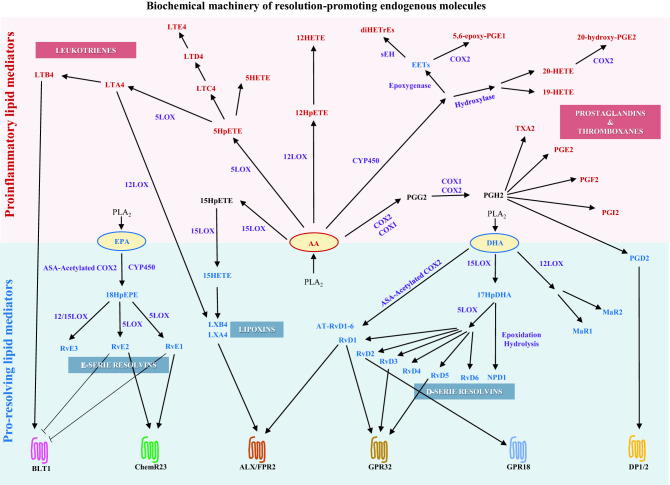
Biochemical machinery of resolution-promoting endogenous molecules. Schematic of biochemical enzymatic reactions from essential fatty acids AA, DHA, and EPA, to the production of their proinflammatory and pro-resolving bioactive lipid metabolites

**Fig. 4:**
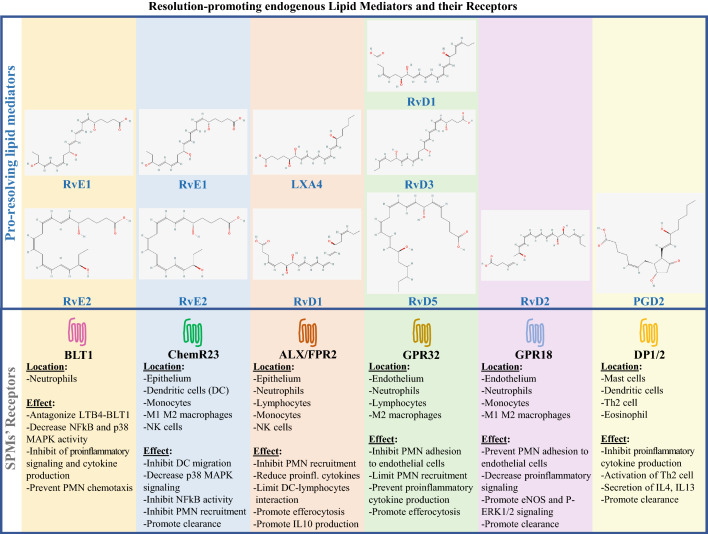
Resolution-promoting Lipid Mediators and their Receptors. Specialized proresolving mediators (SPM), including RvE1, RvE2, RvD1, RvD2, RvD3, RvD5, LXA4, PDG2, and their known receptors. For each SPM, the molecular structure is provided. For each receptor, the main location and the effects of its interaction with corresponding SPM is described. Picture of SPMs’ molecular structure is provided by PubChem’s free database: https://pubchem.ncbi.nlm.nih.gov/

In the present article, the historical evolution of the concept of resolution from 1907 to 2021 is itemized (Fig. 1) and the recent consensus around the biochemical mechanisms of inflammation-resolution is reported (Figs. 2, 3 and 4).

This review focuses on the impact of proresolution therapy in cardiac disease, and discusses evidence of detection of SPMs receptors in cardiac tissue (Table 1), and the relevance of inflammation resolution therapeutic strategies in cardiac disease (Tables 2 and 3), considering the recent preclinical and clinical studies that have evaluated the role of SPMs in myocarditis, myocardial infarction, atherosclerosis, right heart disease, and atrial fibrillation.

**Table 1:** Evidence of SPMs’ receptors expression on cardiac cell types

Cardiac cell type	SPMs’ receptors	Species	Action	References
Cardiomyocytes	ALX/FPR2	Murine	Prevents myocarditis-induced hypertrophy and apoptosis	[33]
	ChemR23	Murine Rat	Modulates p38-MAPK and ERK1/2 pathways, Regulates AKT phosphorylation and apoptosis,	[35, 36, 52]
	GPR18	Rat	Normalizes contractility, prevents ROS accumulation	[34]
Fibroblasts	ALX/FPR2	Murine Rat	Reduces post-MI fibrosis Prevents LPS-induced increase of proinflammatory interleukines levels	[37, 38]
	ChemR23	Rat	Prevents increase in ICAM-1 and VCAM-1 protein levels	[38]
	GPR18	Human	Modulates apoptosis and cell viability in cardiomyoblast	[39]
	GPR32	Murine Rat	Reduces cardiac fibrosis post-MI Prevents atrial fibrosis in right heart disease	[37, 32]
Endothelial cells	ALX/FPR2	Murine	Prevents PMN recruitment Promotes non-phlogistic monocyte infiltration	[42]
	ChemR23			
	GPR18	Murine Human cell	Limits endothelium’s permeability Promotes to adherens-junction integrity	[43]
	GPR32			
Vascular smooth muscle cells	ALX/FPR2	Murine	Blocks PMN infiltration, Promotes non-phlogistic monocyte recruitment	[46]
	BLT1 (blockade)	Human	Prevents VSMC proliferation	[45]
	ChemR23	Human Murine	Attenuates VSMC proliferation and phenotypic alteration	[44, 45]
Immune cells	ALX/FPR2	Murine	Expressed on PMN and macrophages; Prevents PMN infiltration and promote m2-macrophage phagocytosis	[30, 37]
	BLT1 (blockade)	Murine	Expressed on PMN and M1-macrophage; Limited sepsis-induced myocardial scar	[48]
	ChemR23	Human	Expressed on Monocytes and macrophages, Prevented atherogenesis	[47]
	GPR18	Rat	Expressed on PMN, monocytes, lymphocytes, M1 and M2 macrophages; Inhibits PMN infiltration, Promotes M2-macrophage polarization and phagocytosis	[34]
	GPR32	Murine	Expressed on PMN and macrophages, Promotes M2-macrophage polarization, phagocytosis, and clearance	[49]

**Table 2:** Evidence of SPMs’ effects in cardiac diseases

Cardiac disease	SPM	Species	Action	References
Myocarditis	LXA4 BLM-111	Murine	Prevents expression of fibrosis-related genes and oxidative stress markers in ventricular myocardium	[33]
Myocardial infarction	RvE1	Rat	Reduces infarct size, limites PMN infiltration, Decreaes Caspase-3 levels post-I/R	[52]
	RvD1	Murine	Decreases collagen deposition and improves LV function post-MI	[30]
Athrosclerosis	RvD1	Murine	Inhibits necrosis and promotes regression of atherosclerosis	[53]
	RvD2	Murine	Prevent atherosclerosis progression	[54]
	MaR1			
Valvulopathy	RvE1	Human	Reduced phosphate-induced calcification	[56]
		Murine	Prevents aortic valve thickening and dysfunction	
Right heart disease	MAG-DPA	Rat	Prevents Pulmonary Hypertension-induced RV hypertrophy Stimulates RvD5 production in cardiac tissue	[59]
	RvD1	Rat	Improves RHD-induced RV malfunction Attenuates and tricuspid annulus plane systolic excursion	[32]
Congestive heart FAILURE	RvD1	Human	May prevent PMN infiltration, chronic inflammation and CHF progression	[61]
Atrial fibrillation	RvD1	Rat	Prevents RHD-induced atrial fibrosis and inhibits proinflammation and profibrosis-related genes Decreases AF inducibility and AF duration	[32]

**Table 3:** Effect of available antiinflammatory treatments on Resolution and AF incidence

Medication	Beneficial effect on cardiac Events	Interaction with resolution system	Role in af	References
Cox-inhibitors	Blockade of AA-derived proinflammatory mediators	Resolution-Toxic, Inhibit production of AA metabolites involved in activation of resolution and cessation of inflammation	Increase AF risk	[64, 67, 66]
Aspirin	Stops AA-derived proinflammatory metabolomes	Inhibits AA-derived proresolution products Promotes production of AT-Rvs from EPA and DHA	Do not improve AF incidence	[52, 70]
Glucocorticoids	Reduce production of proinflammatory biomarkers	Promote M2-macrophage polarization and clearance	May increase AF risk	[75, 77]
Colchicine	Decreases the risk of ischemic cardiac events (COLCOT, LoDoCo, LoDoCo2 trial)	Inhibits NLRP3 inflammasome, IL1β and IL18 Prevents PMN recruitment Promotes resolution	Decreases AF risk post-op	[78–80, 82]
OMEGA-3 PUFAs	Controversal effects on cardiovascular disease	Reduce-IT: Icosapent ethyl may promote some resolution mechanisms to reduce cardiovascular risk	Increase AF risk	[84]
		Strength: may not promote resolution	Increase AFrisk	[85]
NLRP3-, IL1β and IL6 inhibition	Inhibit NLRP3 assembly and activation Reduce IL1β production (CANTOS trial: Canakinumab) Reduce IL6 levels (RESCUE trial: Ziltivekimab) Decrease biomarkers of inflammation Decrease cardiac fibrosis	Promote lipid-mediator class switching and resolution	Not tested	[95, 99, 100]
		RvD2 prevents NLRP3 activity	Not tested	[98]
		RvD1 NLRP3, ASC and CASP1 expression in RA	Prevents AF inducibility	[32]

## Concept of inflammation resolution

### Historical timeline of knowledge

Inflammation is a normal reaction in response to tissue damage or infection [4]. The essential role and physiological purpose of inflammation are to repair the tissue and to restore its homeostatic function.

One of the first reports of resolution of inflammation is from Eugene L. Opie from the Rockefeller Institute of Medical Research of New York in 1907 [5]. In this article, the author described the role of leukocytes’ enzymatic activity in the resolution of fibrinous pleurisy experimentally induced in dogs. He observed that during the first stage (initiation phase) of inflammation from day 0 to day 3, the animals were inert and fluids with polynuclear leukocytes (PNL) were abundant in the pleural cavity. Then a second phase, the resolution of inflammation (from day 3 to day 5), was characterized by degeneration and disappearance of PNL and fluids, persistence of mononuclear cells, and recovery of the animal’s health [5].

In 1970, Taber’s Cyclopedic Medical Dictionary defined resolution as the decomposition, absorption, or breakdown of the products of inflammation, and the termination of inflammation and return to homeostasis. The molecules and/or cells involved in disappearance of inflammation and/or its dispersion were defined as resolvents [6, 7].

During the 1980s, resolution of inflammation was described as well-orchestrated cellular events from the activation of vascular endothelial cells, and recruitment of polymorphonuclear leukocytes (PMN) in the initiation phase [8], to the clearance of cellular debris and dead PMN cells by macrophages in the resolution phase [9]. The endogenous roles of eicosanoids (Prostaglandins [PGs], thromboxanes [TXs], leukotrienes [LTs]), cytokines, and reactive oxygen species (ROS) on proinflammatory response were discovered and associated with initiation and efferocytosis via leukocyte stimuli, cellular chemotaxis and recruitment, phagocytosis, and induced apoptosis [10, 11].

In the ‘90 s, Serhan and collaborators discovered that lipoxins (LXs), could stop PMN recruitment and attenuate fibrosis [12–14]. Resolution was then described as an active reaction to terminate acute inflammation, and that chronic inflammation may be a consequence of failed resolution process [15]. Antiinflammatories such as glucocorticoids were shown to inhibit the production of proinflammatory products from arachidonic acid (AA) metabolism by inhibition of phospholipase A2 (PLA2) and COX2 enzymes [16]. COX2 inhibitors were also developed to efficiently inhibit the production of PGs, TXs, and LTs but also LXs, which may explain why their use was associated with complications and side effects [17, 18].

Most recently, during the first decade of the 2000s, Serhan and colleagues discovered that non-toxic endogenous autacoids called resolvins (Rvs) are implicated in the active process of resolution [19]. Serhan and collaborators have described that omega-3 polyunsaturated fatty acids (n-3 PUFAs) are metabolized by enzymes such as COX-2 and 5-LOX to produce these bioactive mediators [20]. Hence, eicosapentaenoic acid (EPA) is a precursor to the E-series resolvins (RvE1, RvE2, RvE3), and eicosahexaenoic acid (DHA) is a precursor to the D-series resolvins (RvD1-6), neuroprotectin D1 (NPD1), and maresins (MaR1) [20]. They have also observed that low-dose aspirin can trigger the biosynthetic production of a variety of analog forms of these lipid mediators, called aspirin-triggered resolvins (AT-Rvs) via EPA and DHA [20].

The omega-3-derived promotors of resolution are called specialized pro-resolving mediators (SPMs) [21]. Moreover, the discovery of specific receptors of SPMs (i.e., ALX/FPR2 receptor of LXA_4_ and RvD1; GPR32 receptor of RvD1; ChemR23 receptor of RvE1) supports the concept that resolution is an active process mediated by complex intracellular signaling in response to inflammation, to promote homeostasis and prevent chronic inflammation [22] (Fig. 1).

### Consensus: definition and chronology of events from acute inflammation to resolution

Acute inflammation is characterized by the succession of three major phases: the initiation, lipid-mediators class switching, and resolution. Resolution can be defined as the succession of active processes involving endogenous specialized pro-resolving mediators (SPMs), aiming to terminate inflammation and restore normal functions [23].

In response to chemical or physical deleterious stimuli from infection or injury, the attacked tissue releases pathogen-associated molecular patterns (PAMPs) or damage-associated molecular patterns (DAMPs) that interact with pattern recognition receptors, such as toll-like receptors (TLRs), expressed on the affected cell membrane*.* TLRs activate intracellular pathways including mitogen-activated protein kinases (MAPK) pathways, nuclear factor kappa-light-chain-enhancer of activated B cells (NF-κB). NFkB mediates the activation of expression of various genes involved in the activation of inflammatory processes. Among them, NFkB promotes the assembly and activation of NLRP3-inflammasome and accentuates the secretion of proinflammatory cytokines (IL1β, IL6, IL18), and chemokines (CXCL1/2) involved in the initiation of the inflammatory response [24].

AA is metabolized via CYP450, cyclooxygenase-2 (COX2) and 5-, 12-, and 15-lipoxygenase (LOX) into proinflammatory lipid-mediators including thromboxanes (TXA_2_: vasoconstriction), prostaglandins (PGs: vasodilation, lymphocytes proliferation), and leukotrienes (LTs: vasopermeability, chemotaxis) (Fig. 2) to install the cardinal signs of initiation of inflammation, namely: warmness, redness, nodule, and ache [25].

Apoptotic neutrophils start to produce endogenous SPMs such as lipoxins (LXA_4_ and LXB_4_) [26]. This pro-resolution signal marks the end of the initiation phase of acute inflammation, attenuation of the cardinal signs of inflammation, and the beginning of lipid-mediator-class switching to activate the resolution phase [27] (Figs. 1 and 2).

Circulating essential n-3 PUFAs (EPA and DHA) are accumulated in the damaged tissue via edema to be enzymatically metabolized by COX2 and 5/12/15-LOX and converted into bioactive SPMs. Lipoxins (LXs) inhibit chemotaxis of further PMN and stimulate the nonphlogistic recruitment of monocytes at the site of injury. SPMs, including LXs, Rvs, maresins, and/or protectins are produced and accumulated in the injured tissue. The SPMs stimulate the cessation of neutrophil infiltration in the inflamed area, and they inhibit proinflammatory cytokines-signaling [28]. SPMs promote the differentiation of monocytes into anti-inflammatory (M2) macrophages [29]. SPMs activate efferocytosis and clearance by stimulating M2-macrophage phagocytosis of apoptotic neutrophils and cellular debris. M2-macrophages produce anti-inflammatory cytokines (IL10) and more SPMs to promote resolution. Resolution of inflammation results in tissue healing, relief of pain, remission of fever, and regeneration of normal structure and function [23] (Figs. 1 and 2).

When the tissue fails to activate the lipid-mediator-class switching which promotes SPMs production and resolution, proinflammatory signaling will persist and abnormally stimulate further recruitment of PMN and perpetuation of the inflammatory status. This may promote chronic inflammatory profile, development of fibrosis, and loss of function. However, mechanisms or therapeutic strategies promoting bioavailability and accumulation of SPMs in chronically inflamed organs may activate resolution, wound healing, and restitution of normal function and structure [29, 30] (Figs. 1 and 2).

## Specialized pro-resolving mediators’ receptors in cardiac cells

The presence of SPM receptors is well described in lung, brain, kidney, eye, or liver tissue [29]. Few data are available about their expression in the heart. Some studies on murine models of ischemia–reperfusion reported the presence of SPM receptors in ventricle [30, 31]. Recently, ChemR23, GPR32, ALX/FPR2, BLT1, GPR18, and GPR37 were detected in rat atrial tissue [32]. To our knowledge, little is known about the expression of SPM receptors on isolated atrial cardiomyocytes and atrial fibroblasts.

### SPM receptors on cardiomyocytes

The expression of SPM receptors has been evaluated on cardiomyocytes from rat, murine and human heart tissue. In a mouse model of autoimmune myocarditis, BML-111, an analog of LXA_4_, prevented cardiomyocyte death in vitro. This study suggests that under inflamed conditions, proresolving stimuli may promote cardiomyocyte’s expression of ALX/FPR2, the specific receptor of LXA_4_ [33]. GPR18, the specific receptor of RvD2 was detected on ventricular cardiomyocytes in rats. GPR18 activation ameliorated ventricular function [34]. ChemR23, also known as Chemerin Chemokine-Like Receptor 1, is the receptor of RvE1 and RvE2. ChemR23 was detected on rat ventricular cardiomyocytes [35] and murine atrial cardiomyocytes [36]. ChemR23 activation was shown to mediate cardiomyocyte apoptosis [36] (Table 1 and Figs. 3 and 4).

### SPM receptors in cardiac fibroblasts

The activity of SPM on fibroblast’s proliferation and differentiation have been reported on diseases associated with fibrosis-induced malfunctions, including neurodegenerative diseases or aneurysm [29]. In the heart, studies from Ganesh Halade’s group revealed that RvD1 treatment reduced ventricular fibrosis in myocardial infarction [30, 37]. These data suggest that cardiac fibroblasts may express RvD1 receptors GPR32 and/or ALX/FPR2. Consistent with this, in a rat model of monocrotaline-induced right heart disease, RvD1 reduced atrial fibrosis and atrial fibrosis-related markers POSTN, TGFβ3, COL1A1, COL3A1, and ACTA1 as detected by microarray assay, RT-qPCR, and Western blot [32]. In a recent study, ALX/FPR2 and ChemR23 were detected on isolated ventricular fibroblasts. RvD1 treatment prevented lipopolysaccharide (LPS)-induced increase in IL6, MCP-1, and TNFα. The authors observed that RvD1 and RvE1 prevented the adhesion of mononuclear cells to cardiac fibroblasts [38]. Recently GPR18 was detected on cardiomyoblasts from patients with heart failure. GPR18 expression may be increased in response to injury, and its inactivation may promote heart failure chronicity [39] (Table 1, Figs. 3 and 4).

### SPM receptors on other cardiac cell types

Coronary vessels (arteries and veins) are essential in cardiac physiology to irrigate the heart. Blood supplies the heart muscle with oxygen, nutrients, and immune cells (in case of injury or infection) via the coronary arteries and is drained via the coronary veins [40]. The coronary vessels are made of an inner thin layer of endothelial cells, the intima; a thicker layer made of several concentric sheets of vascular smooth muscle cells (VSMC), the media; and an external layer comprised of elastin and collagen fibers, the adventitia [41] (Table 1, Figs. 2, 3 and 4).

#### SPM receptors on cardiac endothelial cells

Cardiac endothelial cells (CEC) play an important role in the initiation and resolution phases of inflammation. CEC are sensitive to chemotaxis stimuli, as they are in direct contact with blood. They can modulate the intima’s permeability to allow or limit immune cells infiltration in the myocardium. Hence, CEC have been shown to express ALX/FRP2 and ChemR23 which signaling are important in the CEC-controlled cessation of PMN recruitment and the activation of non-phlogistic monocyte infiltration during LM-class switching [42]. It has been shown that EC also express GPR18 and GPR32 that are activated to attenuate EC permeability and promote EC adherens-junction integrity during inflammation [43] (Table1).

#### SPM receptors on cardiac vascular smooth muscle cells

Intimal hyperplasia is a major clinical problem causing coronary artery bypass graft failure due to proliferation and migration of VSMC in the intima of coronary vessels, leading to their progressive obstruction. In a recent study involving a rat model of intima hyperplasia, ChemR23 was detected on VSMC. ChemR23 deletion was associated with progression of intimal hyperplasia and alteration of VSMC phenotype [44]. Consistent with these results, patients with coronary artery disease (CAD) treated with EPA and DHA had higher plasma levels of RvE1 and lower levels of LTB_4_ compared to untreated CAD patients. This was associated with coronary artery plaque regression [45]. The sensitivity of VSMC to LTB_4_ suggests that these cells may express its specific receptor BLT1 [46]. VSMC have also been shown to express ALX/FPR2. VSMC modulates the permeabilization of the artery wall during initiation and resolution of inflammation via activation of ALX/FPR2 which promotes vasorelaxation, cessation of PMN recruitment and augmentation of monocyte infiltration across the coronary vessels wall [46] (Table 1, Figs. 2, 3 and 4).

#### SPM receptors on immune cells in heart tissue

SPMs’ receptors are well described on immune cells [29]. In this section, the review focuses on reports that assessed the expression of SPMs’ receptors on immune cells in the context of cardiac tissue only. In a murine model of myocardial infarction, ALX/FPR2 was expressed on PMN and macrophages. ALX/FPR2 activation limited myocardial scar after myocardial infarction [30, 37]. In patients with CAD, ChemR23 was detected on monocytes and macrophages [47]. BLT1 was shown to be expressed on the surface of PMN, and pro-inflammatory macrophages (M1-macrophages). The authors observed that BLT1 inhibition was associated with amelioration of sepsis-induced myocardial injury [48]. GPR18 was shown to be expressed on PMN, monocytes, lymphocytes, M1- and anti-inflammatory (M2)-macrophages. GPR18 activation promoted inhibition of PMN infiltration, M2-polarization and phagocytosis, and M1-macrophage apoptosis [34]. GPR32 has been proven to be expressed on human PMN and macrophages to promote M2-macrophage polarization and phagocytosis of apoptotic PMN. GPR32 activity via RvD1 is associated with clearance, efferocytosis, and resolution [49, 50] (Table 1 and Figs. 3 and 4).

## Evidence of specialized pro-resolving mediators in cardiac diseases

### Myocarditis and cardiac inflammation

Myocarditis is a progressive inflammation status of the myocardium in response to infection, cardiotoxic insults, or autoimmune disease [1]. Myocarditis is characterized by an unresolved inflammatory profile, cardiac cell death, and oxidative stress, which can lead to severe dilated cardiomyopathy and heart failure [1].

The role of LXA_4_ analog BML-111 has been evaluated on a murine model of experimental autoimmune myocarditis (EAM). Myocarditis was induced in 8-week-old female BALB/c mice by subcutaneous immunization with 350 µg of cardiac myosin in a 1:1 emulsion with Complete Freund's Adjuvant containing 5 mg/mL of Mycobacterium tuberculosis H37RA. Control animals received an equivalent volume of saline. BML-111 (1 mg/kg/d) was injected from day 7 to day 21. All animals were sacrificed at day 21. Compared to control, EAM was associated with statistically significant increased ventricular myocardium expression of inflammation related genes *Il1b*, *Il6*, and *Tnfa*; fibrosis-related genes *Tgfb1*, *Col1a1*, and *Col3a1*; and oxidative stress markers *8-OHdG* and *Keap1*. BML-111 treatment significantly decreased and normalized the level of expression of these genes (except *Il6* and *Col3a1*). The LXA_4_ analog also prevented EAM-induced cardiomyocyte apoptosis, LV hypertrophy, and reduction of LV ejection fraction and fraction shortening. The authors proposed that BLM-111 may play its cardioprotective effects by activating the CaMKK2/AMPK axis to promote activation of antioxidant *Nrf2* pathway [37] (Table 2).

### Myocardial infarction and cardiac fibrosis

Myocardial infarction (MI) is characterized by LV ischemia and persistent irreversible collagen deposition promoting LV loss of function and progression of heart failure [51]. The role of RvE1 was evaluated in an in vivo model of LV ischemia/reperfusion (I/R). Male Sprague–Dawley rats were subjected or not (sham), to the ligation of the left anterior descending coronary artery (LAD). At 28 min post-ligation, the animals received a single intravenous injection of vehicle or RvE1 at 0.03, 0.1, or 0.3 mg/kg. At 30 min post-ligation, ischemia was stopped by releasing the coronary artery snare to allow reperfusion for 4 h. Compared to rats with I/R-alone; RvE1 treatment did not improve the mean blood pressure post-I/R. However, the infarct size was significantly smaller when treated with RvE1 at 0.1 and 0.3 mg/kg. The mean leukocytes infiltration was reduced in the post-ischemic area in RvE1-treated rats (0.3 mg/kg) compared to I/R-alone. Myocardial levels of Ser^473^ phospho-Akt, Thr^308^ phospho-Akt, and phospho-ERK1/2 were significantly increased in I/R animals compared to sham. RvE1 treatment dose-dependently increased these levels compared to I/R-alone without affecting total Akt or total ERK1/2 levels. Moreover, although caspase-3 was significantly increased in I/R rats compared to sham, RvE1 significantly decrease its level of expression in the infarct area when injected at 0.1 and 0.3 mg/kg, 28 min post-I/R [52].

The inflammation resolving response of RvD1 treatment was studied in a mice model of myocardial infarction (MI) using permanent LAD. Infarcted C57BL/6 J mice were treated or not with RvD1 (3 µg/kg/d) 3 h post-MI. The animals were sacrificed 1 day or 5 days post-MI. RvD1 prevented MI-induced LV hypertrophy and attenuated MI-induced reduction of fraction shortening. RvD1 treatment significantly enhanced the expression of ALX/FPR2 in the infarcted area at day 5 post-MI. RvD1 reduced M1-macrophages density, oriented M2-macrophage polarization, and promoted pro-resolution clearance in the LV at day 5 post-MI as shown by decreased expression of *Tnfα*, *Il6*, *Ccl2*, and *Il1β* and increased expression of *Mrc-1*, *Arg1*, and *Ym-1*. Moreover, RvD1 decreased collagen deposition and ECM gene expression thereby improved LV healing and function post-MI [30] (Table 2).

### Atherosclerosis and cardiac ischemia

Chronic unresolved inflammation participates in the development and aggravation of atherosclerosis. Recent reports have attempted to understand the mechanisms by which failed resolution may promote atherosclerosis. It has been shown that in human, 5-LOX-derived SPM levels were lower in vulnerable atherosclerotic plaque compared to stable lesions. The same study reported that administration of RvD1 reduced necrosis and collagenase while promoting efferocytosis, in a murine model of chronic atherosclerosis [53]. Atherosclerosis progression was prevented by RvD2 and MaR1 repetitive treatment in mice [54]. Pirault and Bäck reviewed the role of four SPMs receptors (ALX/FRP2, GPR32, GPR18, and ChemR23) in atherosclerosis, aortic aneurysm, and ischemia/reperfusion. They reported that activation of these receptors may promote cardiovascular protection via activation of P-ERK1/2 and eNOS activity. Inhibition of these specific SPMs’ receptors provoked inhibition of p38 MAPK signaling and reduction of proinflammatory cytokines production in PMN, macrophages, vascular smooth muscle cells, and cardiomyocytes [42].

These data suggest that atherosclerosis is characterized by an abnormal decrease in SPM/[proinflammatory LTs + PGs] ratio. Proresolving treatment may promote resolution and prevent atheroprogression (Table 2 and Fig. 2).

### Valvulopathy and cardiac calcification

Aortic valve stenosis (AVS) is the most common valve disease characterized by thickening and calcification of the aortic valve leaflets (AVL) causing progressive narrowing and malfunction of the aortic valve (AV) [55].

The role of n-3 PUFAs and their derived specialized proresolving mediators have been evaluated on the development of AVS in human tricuspid aortic valves from patients who underwent AV replacement surgery, and in transgenic mice with AVS [56]. It has been shown that EPA and DHA, as well as RvE1 and RvD3 were decreased, whereas LTB_4_ was increased, in calcified compared to non-calcified human AV. RvE1 reduced phosphate-induced calcification in human valvular interstitial cells (VICs) in vitro. In transgenic mice, targeted deletion of ChemR23 induced increased thickening and malfunction of the aortic valve after aortic valve wire injury. The activation of the n-3 PUFA/RvE1/ChemR23 axis promoted M2 macrophage polarization, inflammation-resolution, and AV cusp motion while preventing AVL thickening and calcification [56] (Table 2).

### Right heart disease

Various inflammatory diseases such as congenital cardiac disease, chronic obstructive pulmonary disease, pulmonary hypertension, are associated with right heart remodeling characterized by RV hypertrophy, RH dilation, RV systolic pressure, tricuspid regurgitation, and atrial dilation [57, 58]. The impact of inflammation resolution on the incidence of these diseases is suggested, but little is known on the underlying pathophysiological mechanism. Recently the role of inflammation resolution has been tested in a rat model of right heart disease (RHD)-induced AF. RHD has been induced in Wistar rats by a single injection of 60 mg/kg of monocrotaline *i.p.* Control animals received an equivalent dose of vehicle. Starting one day before MCT administration, the intervention group received daily doses of 2 µg/kg/d of RvD1 for 3 weeks. RvD1 significantly prevented RHD-induced increase in P wave duration, RR and QT intervals, and tricuspid annulus plane systolic excursion [32]. Previously, the role of MAG-DPA (docosapentaenoic acid monoacylglyceride), a synthetic metabolic precursor of EPA and DHA has been tested in an MCT model of PH-induced RHD in rats. MAG-DPA was administrated at 231 mg/kg/d during 3-weeks. MCT-induced increases in RV weight and wall thickness were prevented by MAG-DPA treatment. MAG-DPA stimulated metabolic production of RvD5 in cardiac tissue [59]. These data suggest that proresolving treatment may prevent accentuation of inflammation-induced cardiac structural and functional remodeling in RHD (Table 2 and Fig. 2).

### Congestive heart failure

Congestive heart failure (CHF) is characterized by a typical inflammation profile [60]. A recent report supports the concept that alteration of resolution of inflammation is associated with chronicity of heart failure [61]. This study involved 50 patients: 27 had CHF and 23 were healthy. CHF patients showed lower levels of leukocytic 15LOX activity, D-series resolvins, and RvD1 receptor GPR32 compared to healthy subjects. The authors concluded that altered proresolution mediators may promote chronicity of inflammation and progression of CHF [61] (Table 2 and Fig. 2).

### Atrial arrhythmia

Mounting evidence suggest that unresolved inflammation may promote atrial fibrosis and development of atrial arrhythmogenic substrate leading to enhanced AF susceptibility [62]. The role of RvD1 has been tested in AF prevention. In a rat model of RHD, RvD1 daily treatment attenuated RHD-induced RA fibrosis. RvD1 promoted reduction of density in M1-macrophages and increased M2-macrophages in RA. RvD1 prevented RHD-induced overexpression of proinflammatory markers (CXCL1/2), NLRP3-inflammasome components (ASC, CASP1, IL1β), and fibrosis-related agents (COL3A1, TGFβ3). Although no changes were observed in ALX/FPR2 expression, RvD1 increased ChemR23 expression in RA compared to RHD-only and control animals. RvD1 also prevented RHD-induced RA electrical conduction abnormalities. These right atrial effects of RvD1 were associated with significant prevention of RHD-induced increase in atrial tachyarrhythmia susceptibility [32, 63] (Table 2 and Fig. 2).

## SPMs vs traditional antiinflamatory treatments in cardiac diseases

### Non-steroidal antiinflammatory drugs and SPMs in cardiac diseases and AF

#### COX-inhibitors are resolution-toxic

The normal process of acute inflammation is regulated by bioactive lipid mediators produced from the well-orchestrated sequence of enzymatic interactions of COX1/2 and 5/15/15LOX with AA, EPA, DHA [29]. The highly selective COX2 inhibitors are efficient to block the production of PGs, LTs, and TXs from AA. However, they can cause serious cardiovascular adverse effects [64]. The inhibition of COX2 by COX2-inhibitors is nonselective. Hence, important metabolite products involved in the resolution process (PGE_2_, PGD_2_) are also blocked. In a randomized trial involving 2035 patients, use of celecoxib was associated with a dose-dependent augmentation of mortality from cardiovascular causes, myocardial infarction, heart failure and stroke [65]. Celecoxib has been shown to significantly increase P-wave duration in patients with inflammatory arthritis suggesting that this medication caused atrial remodeling that predisposed these patients to AF [66]. In a 9-year retrospective study on 32,602 patients, the use of COX2 inhibitors was associated with an increased risk of AF and atrial flutter [67] (Table 3 and Figs. 3 and 4).

#### Potential aspirin-triggered resolution

Aspirin is a traditional non-steroidal antiinflammatory drug (NSAID) that acetylates COX2 and stops the production of PGs and LTs from AA. In this manner, aspirin attenuates the LM-class switching and some resolution process mediated by PGE_2_ and PGD_2_. Paradoxically, aspirin-induced acetylated COX2 remains active for enzymatic interactions with EPA and DHA leading to production of aspirin-triggered (AT) resolvins (AT-Rvs). It is unclear whether aspirin-triggered production of SPMs homologs and complete inhibition of PGs would have beneficial outcomes or not for the host. In cardiovascular disease, low-dose aspirin (80 mg and under) is recommended for secondary prevention, when the patient is between 40 to 70 years of age, at high risk of cardiovascular disease and at low risk of bleeding, with a history of coronary disease, stroke, or heart attack. However, aspirin is not recommended as a primary preventive approach for healthy patients [68]. Aspirin has been used in AF treatment as an anticoagulant in some patients [69], but chronic use of aspirin was not associated with diminution of AF incidence [70] (Table 3 and Figs. 3 and 4).

### Impact of glucocorticoids on resolution in cardiac diseases and AF

Glucocorticoids may appear as ideal medications in the treatment of inflammation, particularly cardiac inflammation, as they promote reduction of proinflammatory biomarkers (initiation phase) and stimulate nonphlogistic monocyte polarization into M2-macrophage, efferocytosis, and clearance (resolution phase) [71–73]. In a prospective study, 138 patients that underwent catheter ablation were randomly assigned to two groups: control and steroid treatment. Short-term steroid therapy decreased the incidence of early recurrence of AF compared to control. No efficacity was observed on late recurrence of AF [74]. In a clinical trial involving 4494 patients; dexamethasone did not prevent postoperative AF [75]. Glucocorticoids are known to be associated with various adverse effects and risk of complications [76]. A recent review suggests that their use may increase the risk of atherosclerosis, and atrial arrhythmias [77] (Table 3).

### Other anti-inflammatory targets in resolution of cardiac diseases and AF

#### Colchicine

Colchicine is a well-known antiinflammatory drug with beneficial effects in pericarditis. The mode of action of colchicine suggests beneficial effects to promote resolution of inflammation. Colchicine inhibits NLRP3 inflammasome activation, inactivates NFkB expression, decreases TNFα expression, reduces the release of the pro-inflammatory cytokines IL-1β and IL-18, prevents PMN chemotaxis, and promotes cessation of inflammation [78]. In a retrospective study on 1412 patients, colchicine administration significantly reduced AF incidence after cardiac surgery [79]. Recently, in the COLchicine Cardiovascular Outcomes Trial (COLCOT), low dose colchicine (0.5 mg daily) has been demonstrated to significantly reduce the risk of ischemic cardiovascular events in 2366 MI patients compared to 2379 MI patients treated with placebo [80]. In the Low-dose colchicine trial (LoDoCo) involving 532 patients with chronic coronary disease, daily administration of 0.5 mg colchicine decreased the risk acute cardiovascular events compared to patients treated with placebo [81]. In the LoDoCo2 trial, recently published in 2020, 5522 patients were randomized to receive either colchicine (2762) or placebo (2760). The incidence of spontaneous myocardial infarction, cardiovascular death or ischemia-driven coronary revascularization were significantly lower in patients treated with colchicine compared to placebo [82] (Table 3).

#### Omega-3 PUFAs

During the last two decades, EPA and DHA were central subjects of controversy. Their antinflammatory and antiarrhythmogenic properties have been demonstrated, but no clinical trials confirmed their efficacy to prevent AF [83]. Recently, in the multicenter study REDUCE-IT (Reduction of Cardiovascular Events with Icosapent Ethyl–Intervention Trial), Deepak L. Bhatt and colleagues observed that daily administration of icosapent ethyl (IPE) could decrease the risk of ischemia, myocardial infarction, and cardiovascular death [84]. These results were opposed to the STRENGTH trial results in which combination of EPA and DHA did not show significant benefits [85]. In terms of arrhythmia, these studies suggested that AF risk was increased as 2.2% patients in the omega-3 group vs. 1.3% in the placebo group induced AF in the STRENGTH trial, while 5.3% patients in the IPE group vs. 3.9% in the placebo group induced AF in the REDUCE-IT trial. Regarding the biochemical system involving EPA and DHA in the resolution process, it is suspected that most of trials involving these n-3 PUFAs did not consider promoting their enzymatic metabolism into bioactive compounds [29, 86]. The present review supports the idea that a new paradigm must be adopted in omega-3 treatments considering their level of degradation after administration, their optimized interaction with specific enzymes (5/12/15LOX) and the successful production of their bioactive proresolving metabolites (Table 3 and Fig. 3). Supporting the hypothesis that therapy promoting optimization of SPM production must be prioritized instead of simply administrating Omega-3 PUFAS or inhibiting inflammatory compound productions, the STABILITY trial (STabilization of Atherosclerotic plaque By Initiation of darapLadIb TherapY) involving 15,828 patients revealed that compared to placebo, darapladib (an inhibitor PLA_2_) did not prevent cardiovascular events in patients with coronary heart disease [87]. As previously described, PLA_2_ is at the top of the metabolic cascade of production of either proinflammatory biomarkers from AA and proresolution mediators from EPA and DHA. This review suggests that inhibition of PLA_2_ may prevent production of inflammatory signals but also SPM, which may explain no beneficial effects against cardiovascular events in the STABILITY study (Figs. 2 and 3).

#### NLRP3-inflammasome, IL1 and IL6 INHIBITION

Various chronic cardiac inflammatory diseases, including AF, have in common the overexpression of NLRP3 inflammasome-related genes and components [88–92]. The NLRP3 inflammasome senses PAMPs and DAMPs signals to promote proinflammatory response to infection or injury by stimulating secretion of proinflammatory interleukins such as IL1β, IL6, and IL18 [93, 94]. Genetic deficiency of NLRP3 was associated with reduced expression of proinflammatory cytokines (TNFα, IL1β, IL6), reduced secretion of proinflammatory PGE_2_ and LTB_4_, and increased expression of LXA_4_ and LXB_4_, which suggests that presence of NLRP3 may negatively influence the LM-class switching and prevent resolution process during acute inflammation [95]. In the CANTOS (Canakinumab ANtiinflammatory Thrombosis Outcome Study) trial involving 10,061 patients with myocardial infarction and high-sensitivity C-reactive protein level of ≥ 2 mg per liter, specific inhibition of IL1β by the monoclonal antibody canakinumab resulted in a significant reduction in the risk of cardiovascular events compared to patients treated with placebo [96]. In the randomized RESCUE trial, IL-6 inhibition with ziltivekimab in patients at high atherosclerotic risk was associated with reduction of circulating levels of biomarkers of thrombosis and inflammation commonly related to atherosclerosis [97]. Recently the role of SPMs in the inhibition of NLRP3 inflammasome to promote resolution has been tested [63]. The authors observed that RvD2, via its specific receptor GPR18, suppressed expression of pro-IL1β, reduced secretion of IL1β, attenuated ASC oligomerization, and decreased CASP1 activity [98]. In the heart, murine models of inhibition of NLRP3 by MCC950 or specific knockdown showed reduction of cardiac fibrosis, and expression levels of IL1β and IL18 [99, 100]. In a rat model of right heart disease, RvD1 treatment decreased arrhythmogenic atrial remodeling-associated increases in right atrial expression of NLRP3, ASC, CASP1, and AF inducibility [32, 63] (Table 3).

## Reported limits about SPM’s activity

In a recent study from Giorgio Mottola and collaborators involving a model of rat carotid angioplasty, periprocedural oral administration of RvD1 led to the increased plasmatic concentration of RvD1 3 h post-gavage, which prevented arterial inflammation but did not reduce intimal hyperplasia [101]. In a rat model of right heart disease, daily treatment with RvD1 during 21 days attenuated cardiac fibrosis and inflammation but did not prevent cardiac dilation in response to induced right-sided chronic overload [32]. As previously described in Fig. 3, SPMs are generated via fragile metabolic processes, including oxidation and epoxidation, meaning that SPMs can themselves be metabolized shortly after their production. This character confers an extremely short half-life to the SPMs of about 1 h to 5 h depending on the study [103]. The short half-life of SPMs versus their potential strong pro-resolution effects suggest that strategies to optimize their bioavailability are required [102, 103].

## Summary

Acute inflammation is governed by a complex biochemical system involving bioactive proinflammatory and proresolving lipid mediators. In optimal conditions, the initiation of inflammation must be terminated to optimize the activation of resolution signals promoting clearance and homeostasis. In chronic inflammatory cardiovascular diseases including AF, resolution may have failed to be activated. This review suggests that future therapeutic strategies may target resolution via the maximization of the production of bioactive proresolving mediators and the inhibition of proinflammatory biomarkers, in a spatiotemporal-dependent manner, to prevent or cure cardiac inflammation and AF. In a larger spectrum, identifying the right moment and the right place to tackle inflammation is a significant challenge in the development of new therapies targeting diseases characterized by chronic inflammatory status.

## Abbreviations

AA: Arachidonic acid
AF: Atrial Fibrillation
COX: Cyclooxygenase
IL: Interleukin
LOX: Lipoxygenase
MI: Myocardial Infarction
NLRP3: NOD-like receptor family, pyrin domain containing 3
RHD: Right Heart Disease
Rvs: Resolvin
SPM: Specialized Pro-resolvin mediator
